# Complete Genome Sequence of *Streptococcus mitis* Strain SVGS_061 Isolated from a Neutropenic Patient with Viridans Group Streptococcal Shock Syndrome

**DOI:** 10.1128/genomeA.00259-16

**Published:** 2016-04-07

**Authors:** Varduhi Petrosyan, Michael Holder, Nadim J. Ajami, Joseph F. Petrosino, Pranoti Sahasrabhojane, Erika J. Thompson, Awdhesh Kalia, Samuel A. Shelburne

**Affiliations:** a Graduate Program in Diagnostic Genetics, School of Health Professions, The University of Texas M.D. Anderson Cancer Center, Houston, Texas, USA; b The Alkek Center for Metagenomics and Microbiome Research, Department of Molecular Virology and Microbiology, Baylor College of Medicine, Houston, Texas, USA; c Department of Infectious Diseases, Infection Control and Employee Health, The University of Texas MD Anderson Cancer Center, Houston, Texas, USA; d Sequencing and Microarray Facility, The University of Texas MD Anderson Cancer Center, Houston, Texas, USA; e Department of Genomic Medicine, The University of Texas MD Anderson Cancer Center, Houston, Texas, USA

## Abstract

*Streptococcus mitis* frequently causes invasive infections in neutropenic cancer patients, with a subset of patients developing viridans group streptococcal (VGS) shock syndrome. We report here the first complete genome sequence of *S. mitis* strain SVGS_061, which caused VGS shock syndrome, to help elucidate the pathogenesis of severe VGS infection.

## GENOME ANNOUNCEMENT

Among the different species comprising viridans group streptococci (VGS), *Streptococcus mitis*, which is closely related to *Streptococcus pneumoniae,* is the most frequent cause of bacteremia in neutropenic cancer patients (1). The clinical presentation of *S. mitis* bacteremia in neutropenic patients can vary from mild to severe, for example, VGS shock syndrome. Moreover, invasive *S. mitis* strains are often multidrug resistant (2), which increases the risk of adverse patient outcomes (3). Despite the increasing clinical relevance of *S. mitis* infections, little is known about their pathogenesis.

Here, we report the complete genome sequence of the multidrug-resistant *S. mitis* strain SVGS_061, which was isolated from the bloodstream of a neutropenic-acute myelogenous leukemia patient with VGS-shock syndrome. SVGS_061 was resistant to moxifloxacin (MIC, 4 µg/ml) and tetracycline (MIC, 16 µg/ml) and had intermediate resistance to penicillin (MIC, 1 µg/ml). The SVGS_061 genome was determined using the PacBio SMRT technology (4). A total of 68,561 reads were assembled using the Hierarchical Genome Assembly Process for *de novo* genome assembly. MiSeq short reads were then used to confirm the 2,167,922-bp circularized genome assembly, with 151× average sequencing depth. The assembled genome was annotated with RASTtk (5), which identified 1,986 coding sequences, 59 tRNAs, and a host of intergenic repeat unit of pneumococcus (RUP) (*n* = 15), SPRITE (*n* = 18), and BOX (*n* = 81) repeats that are typically present in *S. pneumoniae* genomes in high density and likely regulate gene expression (6, 7). A putative 96-kb (genome coordinates 1076140 to 1172058) Tn*5253*-like integrative and conjugative element (ICESVGS_061) was identified and was most similar to ICESpn*22664* from *S. pneumoniae* (99% identity over 48% nucleotide overlap). ICESVGS_061 contained several hallmark proteins, including site-specific integrases, type IV secretion, conjugation protein homologs, and Tn*5252* and Tn*916* open reading frames (ORFs) (8, 9). Moreover, the SVGS_61 integrative and conjugative element (ICE) contains *mef* (locusID_AXK38_05275)*, tetM* (locusID_AXK38_05320), and *cat* (locusID_AXK38_05440*)* genes that confer macrolide, tetracycline, and chloramphenicol resistance, respectively. Furthermore, combined CARD (10) and BLAST analyses identified mutations known to confer high-level fluoroquinolone resistance in GyrA (Ser81Phe) (locusID_AXK38_06150) and ParC (Ser79Ile) (locusID_AXK38_06460) (11–13); the genome also harbored the p*mrA* (locusID_AXK38_03855) efflux gene, which is associated with fluoroquinolone resistance (14). Mutations associated with increased penicillin resistance in penicillin-binding protein 1a (PBP 1a) (Val408Leu) (locusID_AXK38_08480), PBP 2b (Gln628Glu) (locusID_AXK38_02845), and PBP 2x (Asn417Lys, Leu510Thr, and Thr513Asn) (locusID_AXK38_08630) were also identified (15).

Homologs of a number of *S. pneumoniae* virulence-associated proteins, such as the capsular proteins encoded by the *cps* operon, cell wall synthesis-associated proteins, lyase (NanA), and amidase (LytC), were identified via the VFDB database (16) search. The capsule is a crucial virulence factor for *S. pneumoniae*. Capsular proteins of SVGS_061 are most closely related to serotype 4F and are most similar to the capsular proteins of *S. pneumoniae* TIGR4 (96% identity over 56% nucleotide overlap). OrthoMCL analysis (17) identified 1,509 orthologs in the *S. pneumoniae* TIGR4 genome. Exotoxins similar to those causing toxic shock in staphylococci or β-hemolytic streptococci were not identified in SVGS_061. The availability of the complete genome sequence of SVGS_061 should help facilitate a better understanding of the VGS shock syndrome resulting from *S. mitis* invasive infection.

### Nucleotide sequence accession number.

The complete genome sequence has been deposited at DDBJ/EMBL/GenBank under the accession no. CP014326. The version described in this paper is the first version.
